# Health care utilization and costs in Saskatchewan's registered Indian population with diabetes

**DOI:** 10.1186/1472-6963-7-126

**Published:** 2007-08-13

**Authors:** Sheri L Pohar, Jeffrey A Johnson

**Affiliations:** 1 Canadian Agency for Drugs and Technology in Health, Ottawa, Canada; 2 Institute of Health Economics, Edmonton, Canada; 3 School of Public Health, University of Alberta, Edmonton, Canada

## Abstract

**Background:**

The prevalence of diabetes in North American is recognized to be higher in Aboriginal populations. The relative magnitude of health care utilization and expenditures between Aboriginal and non-Aboriginal populations is uncertain, however. Our objective was to compare health care utilization and per capita expenditures according to Registered Indian and diabetes status in the province of Saskatchewan.

**Methods:**

Administrative databases from Saskatchewan Health were used to identify registered Indians and the general population diabetes cases and two controls for each diabetes case. Health care resource utilization (physician visits, hospitalizations, day surgeries and dialysis) and costs for these individuals in the 2001 calendar year were determined. The odds of having used each resource category, adjusted for age and location of residence, was assessed according to Registered Indian and diabetes status. The average number of encounters for each resource category and per capita healthcare expenditures were also determined.

**Results:**

Registered Indian diabetes cases were younger than general population cases (45.7 ± 14.5 versus 58.4 ± 16.4 years, p < 0.001) and fewer were male (42.3% versus 53.2%, p < 0.001). Registered Indians were more likely to visit a physician, be hospitalized or receive dialysis than the general population, regardless of diabetes status. Diabetes increased the probability of having used all resource categories for both Registered Indians and the general population. Per capita health care expenditures for the diabetes subgroups were more than twice that of their respective controls and were 40% to 60% higher for registered Indians than the general population, regardless of diabetes status.

**Conclusion:**

Relative to individuals without the disease, both registered Indians and the general population with diabetes had substantially higher health care utilization and costs. Excess hospitalization and dialysis suggested that registered Indians with and without diabetes experienced greater morbidity than the general population.

## Background

There are significant disparities between the health status of Aboriginal and non-Aboriginal populations in Canada, the United States, New Zealand and Australia [1-6]. Aboriginals rate their health status lower and have higher mortality, hypertension, arthritis, heart disease and diabetes rates than the general Canadian population [1,4-12]. Approximately 5% of all Canadians aged 20 years or older are affected by diabetes [13], but the prevalence of diabetes in Aboriginals is approximately 3.6 times higher among men and 5.3 times higher among women compared to non-Aboriginals [8]. The Aboriginal population also experiences excessive diabetes-related morbidity and mortality relative to the general population with the disease [8,14,15]. Rates of macrovascular and microvascular complications are higher and these complications occur after shorter disease duration in Aboriginals than in the general population with diabetes [5].

Diabetes is a chronic medical condition associated with substantial health care costs [16-19]. The costs associated with managing diabetes have risen over the past few decades, in part due to increased disease prevalence, but also due to increased utilization of health care resources [16]. It is anticipated that the burden of diabetes in Canada will continue to grow in the next decade, with the number of individuals with diabetes reaching 2.4 million and health care expenditures reaching $8.14 billion nationally by 2016 [20]. With the high prevalence of diabetes in Aboriginals, health care costs for this group is particularly relevant from the perspective of Canada's publicly funded health care system.

Poor health status and chronic conditions have been associated with increased utilization of health care resources [21-28]. Given the health status of Canada's Aboriginal population with diabetes and the comorbidity rates they experience, it would be expected that Aboriginals would have considerably higher health care resource utilization and costs than general population diabetes cases. However, utilization of health care resources in the Aboriginal population may be hindered by a number of characteristics of this population. Aboriginals are more likely to live rurally, which may limit access to primary care physicians and specialists [29,30]. Lower socioeconomic status and simply being an ethnic minority may further limit access to primary care in this population [2,30]. Limited access to primary care is thought to relate to increased utilization of other categories of health care resources, such as hospitals [10,17,31,32].

Previous estimates of excess cost of diabetes in the Aboriginal population in Canada did not directly compare utilization rates of individual components of overall health care costs [17]. Because utilization of health care resources for some categories of expenditures could potentially be lower and others higher in Aboriginals with diabetes, the relative magnitude of health care expenditures between Aboriginal and non-Aboriginal populations is uncertain. Thus, the objective of this analysis was to compare physician visits, hospitalizations, day surgeries, dialysis patterns and overall health care expenditures according to registered Indian and diabetes status.

## Methods

### Data sources

The linkable administrative databases from Saskatchewan Health, containing information on physician services, hospitalizations, day surgeries, and dialysis for all eligible residents of Saskatchewan, were used in the analyses [33]. These databases capture covered health services for essentially the entire population of Saskatchewan, approximately 1 million people, with no eligibility distinction by socioeconomic status. These data have been used in numerous epidemiologic studies[33] and have been described elsewhere for use in estimating costs of complications in diabetes [19].

### Study population

The National Diabetes Surveillance System (NDSS) criteria [13] were applied to Saskatchewan Health's administrative databases to identify individuals with diabetes between 1991 and 2001. The NDSS case definition identifies individuals with diabetes through utilization of health care services. Individuals are considered to have diabetes if they have two physician visits with a diagnosis of diabetes (ICD-9 code of 250) on two different days within any contiguous 730-day period or one hospitalization with a discharge diagnosis of diabetes (ICD-9 code of 250 from the first three diagnostic fields) [13]. The ICD-9 code of 250 excludes gestational diabetes. Individuals who met the NDSS criteria for diabetes in the years 1989 or 1990 were also identified and were considered prevalent diabetes cases in 1991. Although the NDSS criteria are generally applied to individuals over the age of 20, we included individuals of all ages.

For each diabetes case, two controls were randomly selected from the non-diabetes Saskatchewan population during the cases index year, and who were not subsequently identified as a diabetes case during the follow-up period. Cases and controls were matched on registered Indian status, as identified by Saskatchewan Health. Registered Indians are people who are registered according to the *Indian Act *and, as such, some individuals of Aboriginal ancestry may be excluded using this definition (i.e., Metis, Inuit and First Nations who are not registered would be considered part of the general population). Of those individuals identified between 1991 and 2001, diabetes cases and controls with active Saskatchewan Health coverage in 2001 were included in the study; individuals who died or whose coverage was terminated prior to 2001 were excluded. Due to differential exit rates over the observation period up to 2001, the ratio of cases to controls was no longer 2 to 1.

### Estimation of healthcare resource utilization and costs

Health care services (physician visits, hospitalizations, day surgeries and dialysis) were extracted from service claims recorded during 2001 in the linkable administrative databases of Saskatchewan Health. Costs of physician visits were collected from each claim record. Physician visits included visits to general practitioners, specialists, and out of province physicians, as well as visits to other practitioners who provided insured services, such as optometrists. Due to the nature of billing data in Saskatchewan, capture of visits to salaried and contract physicians was incomplete.

The number of hospitalizations and day surgeries in 2001 was obtained from Saskatchewan Health's hospital separation file, which includes data on all hospital discharges for Saskatchewan Health beneficiaries (including out of province hospitalizations). Each inpatient record contained a resource intensity weight (RIW), calculated by the Canadian Institute of Health Information, and each day surgery contained a Day Procedure Group (DPG weight). The discharge date was used to determine if a hospitalization occurred in the 2001 calendar year. The cost per hospitalization or day surgery was determined by multiplying the RIW or DPG weight by the funding per weighted case for 2001/2002 [18] which was estimated to be ($3,369.77) (personal communication, M. R. Stang; March 4, 2005). Missing RIWs and DPG weights were estimated using an algorithm provided by Saskatchewan Health (based, in part, on length of stay) and mean imputation, respectively.

Physician fee-for-service billing codes were used to identify individuals who were on dialysis. Duration, frequency and patterns of dialysis were used to estimate the duration of time each year that individuals were on hemodialysis or peritoneal dialysis. Duration of hemodialysis was determined for each year by subtracting the earliest hemodialysis code from the last hemodialysis code within a given year. The duration of time between the last hemodialysis code in one year and the first hemodialysis code in the subsequent year was determined. If this period of time was less than 14 days, individuals were assumed to be on hemodialysis until the end of the calendar year and from the beginning of the next calendar year. It is likely that individuals on hemodialysis would receive the procedure three times per week, but not all of these events would have a fee-for-service billing code. It was felt that individuals on ongoing dialysis would have a fee-for-service code at least every two weeks. Thus, throughout a calendar year, hemodialysis was assumed to be ongoing if the average number of days between hemodialysis codes within that year was less than or equal to 14 days. If the duration of time between hemodialysis codes exceeded 14 days on average, the duration of hemodialysis within a given year was estimated from individual dialysis codes. The same algorithm was used to determine the duration of peritoneal dialysis. Annual dialysis costs were calculated by multiplying the proportion of each calendar year on either hemodialysis or peritoneal dialysis by an estimated annual cost of each dialysis modality [34]. The cost estimate from a prospective observational study of patients attending dialysis clinics in Calgary, Alberta was used [34]. We used these authors' estimated annual cost of either hemodialysis or peritoneal dialysis excluding physician services (as these costs were captured in the physician visits costs). Those values were converted to 2001 Canadian dollars for our estimates ($52,719 dollars per year of hemodialysis or $37, 431 dollars per year of peritoneal dialysis).

Prescription drug use was excluded as an expenditure category because these data were not available for registered Indians. Prescription drug benefits for the registered Indian population are provided by First Nations & Inuit Health, Health Canada and are not included in Saskatchewan Health's databases [35].

### Analysis

Health care resource utilization was determined for diabetes cases and controls according to Registered Indians status, thus creating four subgroups: general population diabetes cases, general population controls, registered Indian diabetes cases and registered Indian controls. Individuals in each subgroup were categorized according to whether or not they used each category of health care resources in 2001 (i.e., no encounters versus one or more encounters). The odds of having used each resource category was determined for (1) general population cases compared to general population controls, (2) registered Indian cases compared to registered Indian controls, (3) registered Indian diabetes cases compared to general population diabetes cases and (4) registered Indian controls compared to general population controls. Odds ratios were adjusted for age, sex and location of residence (large urban [>100,000], small urban [5,000–99,999] or rural [<5,000] residence) using logistic regression.

For each category of healthcare resource (other than dialysis) the average number of encounters was determined for each of the four subgroups. For dialysis, we estimated the average number of days in 2001 on each dialysis modality. To adjust for differences in the age distributions in the four subgroups, the average number of encounters or dialysis days for each subgroup was directly age-standardized to the 2001 Canadian population. Confidence intervals (95%) for both crude and age-standardized point estimates were calculated based on the normal distribution given the large sample size. Crude and directly age-standardized per capita healthcare expenditures were estimated for the four cohort subgroups. Again, 95% confidence intervals were calculated based on the normal distribution.

This study was approved by the Health Research Ethics Board, Panel B, at the University of Alberta.

## Results

### Demographic characteristics

A total of 64,079 individuals met the NDSS criteria for diabetes in Saskatchewan from 1991 to 2001, 46,914 of whom had active coverage in 2001. Approximately 11.3% of the diabetes cases were Registered Indians (n = 5,284) (Table 1). Both registered Indian and general population cases were significantly older than their respective controls (Table 1). Registered Indian diabetes cases were younger than general population cases (45.7 ± 14.5 versus 58.4 ± 16.4 years, p < 0.001). The sex distribution of controls was similar for registered Indians and the general population; however, within the diabetes subgroups only 42.3% of registered Indian cases were male, compared to 53.2% of general population cases (p < 0.001). Registered Indians were more likely to live rurally, particularly registered Indian diabetes cases (Table 1).

**Table 1:** Demographic characteristics of diabetes cases and controls according to Registered Indian status

	**Diabetes Cases**		**Controls**	
	Registered Indian	General Population	Registered Indian	General Population

N (%)	5,284 (11.3)	41,630 (88.7)	11,692 (10.6)	98,680 (89.4)
Age – Mean (S.D.) years	45.7 (14.5)^A,B^	58.4 (16.4)^C^	24.3 (15.0)	37.4 (20.6)
Sex – n (%) Male	2,234 (42.3)^A,B^	22,131 (53.2)^C^	5,912 (50.6)	48,623 (49.3)
Residence – n (%) Rural	3,645 (69.0)^A,B^	19,203 (46.1)^C^	6,980 (59.7)	42,847 (43.4)

A p < 0.001 for comparisons between Registered Indian and general populations cases

B p < 0.001 for comparisons between Registered Indians cases and controls

C p < 0.001 for comparisons between the general population cases and controls

### Odds of health care utilization – general population and registered Indian diabetes cases compared to controls

**Figure 1:**
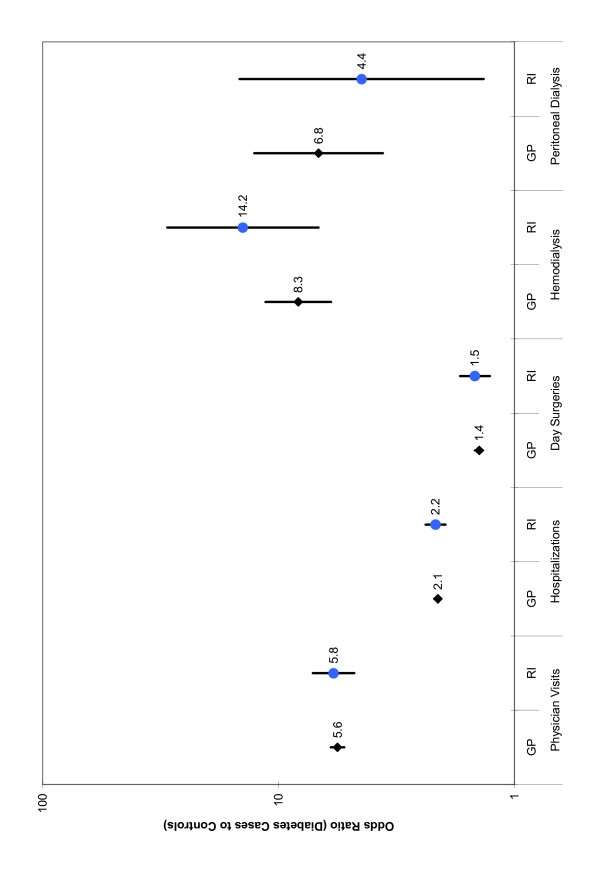
**Health care resource utilization: Adjusted^A ^odds ratios with 95% CI's for registered Indians and the general population (diabetes cases compared to controls)^B^**. A Odds Ratios were adjusted for age, sex and location of residence. B p < 0.05 for all odds ratios.

General population and registered Indian diabetes cases were more likely to have used all four categories of health care resources than their controls in 2001 (Figure 1). The most dramatic difference in utilization was observed for hemodialysis. For the general population, the adjusted probability of having one or more days of hemodialysis in 2001 was 8.6 (95% CI: 6.1 – 11.8) times higher in those with diabetes compared to their controls, while the probability was 14.2 (95% CI: 6.8 – 29.7) times higher in registered Indians with diabetes than their controls. The odds of having peritoneal dialysis were also significantly higher for both general population and registered Indian diabetes cases (Figure 1).

General population and registered Indian diabetes cases were 5.3 (95% CI: 4.9 – 5.6) and 6.2 (95% CI: 5.0 – 7.5) times more likely, respectively, to have had one or more physician visit during the 2001 calendar year than their controls (Figure 1). Hospitalizations were approximately twice as likely to occur in general population and registered Indian diabetes cases compared to controls, while the odds of having a day surgery was 40 to 50% greater in diabetes cases than controls (Figure 1).

### Odds of health care utilization – comparison of general population to registered Indian population for cases and controls

**Figure 2:**
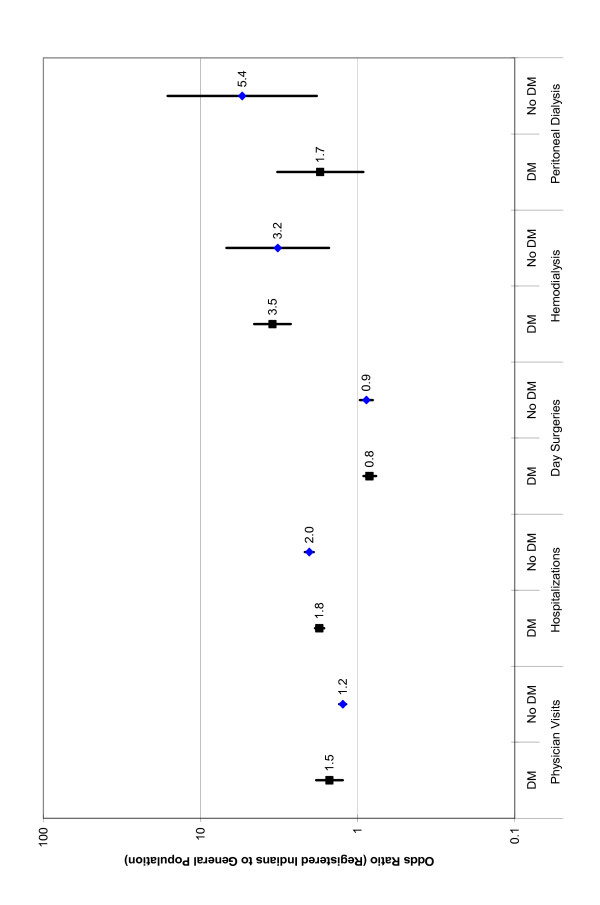
**Health care resource utilization: Adjusted^A ^odds ratios with 95% CI's for diabetes cases and controls (registered Indians compared to the general population)^B^**. A Odds Ratios were adjusted for age, sex and location of residence. B p < 0.05 for all odds ratios with the exception of the comparison of registered Indian to general population diabetes cases for peritoneal dialysis.

Registered Indian diabetes cases and controls were more likely to have a physician visit or hospitalization and were more likely to have received hemodialysis or peritoneal dialysis than general population diabetes cases and controls (Figure 2). However, registered Indians were 10 to 20% less likely to have had day surgery in 2001 (Figure 2). The largest differences between the general and Registered Indian populations for observed for dialysis, with the probability of receiving hemodialysis being approximately three times higher in Registered Indian diabetes cases and controls. Registered Indian controls were 5.4 (95% CI: 1.8 to 16.2) times more likely to have received peritoneal dialysis than general population controls, but for those with diabetes, the probability of receiving peritoneal dialysis did not differ significantly according to Registered Indian status (OR = 1.7; 95% CI: 0.9 to 3.1) (Figure 2).

### Average health care utilization and costs

Average utilization of all categories of health care resources was higher for registered Indian and general population diabetes cases than their respective controls (Table 2), before and after age-standardization. Total health care costs were also higher for both diabetes subgroups relative to their respective controls regardless of registered Indian status (Table 2). Crude per capita health care costs for registered Indians with diabetes were $3,622 (95% CI: $3,336 to $3,908), compared to $875 (95% CI: $801 to $949) for registered Indian controls. Age-standardization decreased the magnitude of the difference, but per capita costs remained several times higher for registered Indians with diabetes. Similarly, costs were more than twice as high in general population with diabetes compared to their controls (Table 2).

**Table 2:** Mean (95% CI) health care resource utilization and costs according to Registered Indian and diabetes status

	**Diabetes Cases Mean (95% CI)**		**Controls Mean (95% CI)**	
	Registered Indians	General Population	Registered Indians	General Population

Average Number of Physician Visits				
Crude	17.3 (16.7 – 17.8)	14.6 (14.4 – 14.7)	6.8 (6.6 – 7.0)	6.4 (6.3 – 6.4)
Age-Standardized	15.0 (14.8 – 15.1)	11.8 (11.8 – 11.9)	7.9 (7.8 – 8.0)	6.4 (6.3 – 6.4)
Average Number of Hospitalizations				
Crude	0.57 (0.54 – 0.61)	0.41 (0.40 – 0.42)	0.16 (0.15 – 0.17)	0.12 (0.11 – 0.12)
Age-Standardized	0.59 (0.56 – 0.63)	0.31 (0.30 – 0.32)	0.24 (0.22 – 0.26)	0.12 (0.11 – 0.12)
Average Number of Day Surgeries				
Crude	0.14 (0.12 – 0.15)	0.22 (0.22 – 0.23)	0.05 (0.05 – 0.06)	0.10 (0.10 - 0.10)
Age-Standardized	0.11 (0.10 – 0.12)	0.15 (0.15 – 0.16)	0.08 (0.07 – 0.08)	0.10 (0.10 - 0.10)
Average Days of Hemodialysis				
Crude	3.73 (2.80 – 4.65)	1.16 (0.98 – 1.34)	0.19 (0.05 – 0.34)	0.09 (0.06 – 0.12)
Age-Standardized	2.60 (2.56 – 2.64)	1.04 (1.02 – 1.05)	0.30 (0.28 – 0.31)	0.09 (0.09 - 0.09)
Average Days of Peritoneal Dialysis				
Crude	0.60 (0.24 – 0.95)	0.29 (0.20 – 0.38)	0.10 (0.0 – 0.20)	0.04 (0.02 – 0.06)
Age-Standardized	0.36 (0.35 – 0.37)	0.39 (0.38 – 0.40)	0.16 (0.15 – 0.17)	0.04 (0.04 - 0.04)
Total Per Capita Costs ($)				
Crude	3,622 (3,336 – 3,908)	3,253 (3,164 – 3,343)	875 (801 – 949)	878 (856 – 901)
Age-Standardized	3,204 (3,201 – 3,206)	2,257 (2,256 – 2,258)	1,395 (1,394 – 1,397)	875 (875 - 875)

Per capita health care costs of the registered Indian population with and without diabetes were considerably higher than the general population, before and after age-standardization. After considering differences in the age distribution of the two populations, per capita health care expenditures were 40% to 60% higher for registered Indians than the general population for both diabetes cases and controls (Table 2).

## Discussion

Health care utilization and costs in Aboriginals deserve attention, not only because of the high prevalence rate of diabetes in this population [36], but also because of potentially inadequate access to the primary care that is essential to appropriate diabetes management [29,30,37]. We found that health care utilization and costs were higher in the registered Indian population than the general population. Further, diabetes itself was an important determinant of health care resource utilization, not only in terms of increased probability of utilization, but also in terms of utilization rates. These trends translated into higher overall health care costs for individuals with diabetes relative to controls and for Registered Indians relative to the general population.

Regardless of registered Indian status, diabetes was associated with excess health care utilization and twice the overall health care costs of controls, an estimate similar to previous studies [16,38]. While registered Indian status and diabetes were both associated with increased health care resource utilization, diabetes appeared to be more strongly associated with health care resource use than registered Indian status alone. This was evidenced by the fact that differences in overall costs were greater according to diabetes status than registered Indian status. Thus, all individuals with diabetes, particularly registered Indians, must have access to the care that they need in order to limit downstream health care costs associated with diabetes and its complications, such as hospitalizations and dialysis.

Past research has demonstrated high hospitalization rates for Aboriginals with and without diabetes relative to the general population, attributed, in part, to inadequate access to primary care[31]. We found that even after considering the differences in age and location of residence, the probability of having one or more physician visit in 2001 was 20% to 60% higher in registered Indian controls and diabetes cases than the general population. The average number of physician visits was also higher for both Registered Indian subgroups. Thus, registered Indian's access to physicians did not appear to be limited relative to the rest of the population. These results are similar to a Manitoba-based study found that Aboriginals had a higher average number of physician visits [4]. An Ontario-based, however, study found that fewer Aboriginals than non-Aboriginals had seen a physician in the previous year [11].

The quantity of visits physician visits does not, however, ensure that the quality or content of the patient-physician interaction or level of care received was the same for registered Indians and the general population. Merely having access to primary care physicians does not guarantee that appropriate or beneficial care is received. The degree to which care is culturally appropriate, for example, may be an important determinant of the outcome of care in Aboriginals [2]. The absolute number of visits is not likely to be an adequate marker for quality of primary care in registered Indians. Thus, additional indicators, such as screening for complications and use of clinical practice guideline recommended preventive therapies during primary care visits, should also be considered in future research.

Higher utilization of physicians, hospitals and dialysis was associated with 40 to 60% higher health care costs for registered Indian diabetes cases and controls than the general population. A previous study found that overall health care costs (i.e., hospital, home care, dialysis and physician costs) were approximately 70% higher in registered Indians with diabetes than the general population with diabetes [17]. The authors attributed the majority of excess costs to higher hospitalization rates in the Registered Indian population. In our present study, it is also likely that differences in hospitalization rates accounted for the difference in overall costs, because hospitalizations represented 75% of total costs in 2001 [39]. The disproportionate morbidity burden and relatively poor health status of this population are thought to contribute to excess hospitalization in this population [4]. In diabetes, complications increase the risk of hospitalization[40,41]. Given the high complications rates in Aboriginals with diabetes[42], it is not surprising that hospitalization rates and subsequent costs would be highest in this group.

While these analyses revealed some interesting trends, a number of limitations should be noted. First, although there is good evidence of validity of the NDSS criteria for identifying diabetes cases, some cases may still be missed [43]. Individuals whose physicians do not use fee-for-service billing, for example, may not meet the NDSS criteria for diabetes unless they are hospitalized for diabetes. This accounts for less than 10% of the approximately 1400 physicians in Saskatchewan[33]. Further, settlements in Saskatchewan's northern health districts are served by fee-for-service physicians and nurse practitioners (NPs). Although both groups may shadow bill, the records were not included in the compilation of the dataset for this project. Thus, it is possible that individuals with diabetes who reside in these areas may be misclassified as controls; it is likely that this misclassification would occur more frequently in the registered Indian than general population. Misclassification in this manner would, however, reduce the magnitude of differences between cases and controls.

Odds ratios were adjusted for location of residence. Prior to 1998, registered Indians were categorized as based on reserve affiliation, rather than last known residence. Thus, there is some possibility of misclassification on this variable if the last known residence was reported prior to 1998. The number of misclassified individuals is not likely to be a substantial given that in order to be included in the analysis, Saskatchewan Health coverage had to be active in 2001.

Resource utilization and cost comparisons between cohorts were limited to four categories of direct costs and therefore underestimate total health care costs for all subjects. We were unable to capture resource utilization and costs managed under global budgets with health regions in Saskatchewan. This would include resources such as emergency department visits, laboratory tests, nurse practitioners, diabetes educators, dieticians, podiatrists, home or long-term care and auxiliary costs of transplants (e.g., transplant coordinators and costs for living donors). The lack of information on prescription drug use for the registered Indian population also limits our comparisons, particularly given that this category represents the second largest component of overall health care costs. Utilization of these resource categories could differ between the registered Indian and general population, which could potentially have an impact on overall cost comparisons. However, hospitalizations remain the strongest driver of overall costs, so it is not clear to what degree overall expenditures would be influenced even if differences in utilization and costs amongst these other resource categories existed.

## Conclusion

Diabetes is a chronic medical condition that is associated with considerable health care costs. Relative to individuals without the disease, both registered Indians and the general population with diabetes had substantially higher health care utilization and costs. Excess hospitalization in registered Indian cases and controls suggested that registered Indians experienced greater morbidity than the general population, regardless of diabetes status. These results highlight the importance of primary and secondary prevention of diabetes and its complications in both populations and provide further evidence of the disproportionate health burden of registered Indians in Canada.

## Competing interests

The authors declare that they have no competing interests.

## Authors' contributions

SLP participated in the design of the study and performed the statistical analysis. JAJ conceived of the study, secured the data, participated in its design and coordination and helped to draft the manuscript. Both authors read and approved the final manuscript.

## Pre-publication history

The pre-publication history for this paper can be accessed here:


